# Cytoplasmic signaling in the control of mitochondrial uproar?

**DOI:** 10.1186/1478-811X-6-4

**Published:** 2008-08-19

**Authors:** Martin Hermann, Andrey Kuznetsov, Manuel Maglione, Julija Smigelskaite, Raimund Margreiter, Jakob Troppmair

**Affiliations:** 1 KMT Laboratory, Innsbruck Medical University (IMU), Innrain 66, A-6020 Innsbruck, Austria; 2 Daniel Swarovski Research Laboratory, Innsbruck Medical University (IMU), Innrain 66, A-6020 Innsbruck, Austria; 3 Center of Operative Medicine, Department of Visceral, Transplant and Thoracic Surgery, Anichstrasse 35, Innsbruck Medical University, A-6020 Innsbruck, Austria

## Abstract

The concept of a pre-emptive strike as a good means to prevent greater harm may be frequently over-stressed in daily life. However, biological systems in a homeostatic balance are prepared to withstand a certain degree of hostile fire by rather passive means. This also applies to the maintenance of cell survival, where a plethora of protective proteins provide safeguard against erroneous activation of death pathways. Apart from these mechanisms active processes are also essential for the maintenance of cellular homeostasis, commonly referred to as survival signaling. Frequently their targets may be mitochondrial, assuring organelle integrity, which is essential for continued energy production and survival. Transient or permanent failures in these cellular defense strategies result in pathophysiological conditions, which manifest themselves e.g. as cancer or ischemia/reperfusion-associated organ damage.

## Review

During times of peace cells are dependent on a balanced crosstalk with their surrounding environment. Survival and proper functioning not only require physical contact with their neighbors, but also depend on a plethora of soluble factors such as hormones or growth factors. The full spectrum of possible cell fates is governed by these mutual and highly dynamic interactions. In the extreme case a dissonance in this "orchestra of life" leads to cell death. However, in order for this to happen a variety of safeguard mechanisms have to fail and cells are normally sufficiently guarded against these threats.

Subversion of intracellular control mechanisms is linked to many pathophysiological conditions. In particular, cancer cells are geared towards cellular autonomy, which is achieved through aberrant activation of intracellular signaling processes. As a consequence these cells are in a preemptive way programmed to withstand hostile conditions and to thrive in a growth and survival limiting environment. Thus cancer provided a useful model for the delineation of mechanisms used to counter potential assaults. Its analysis taught us that tumors in a unique way are equipped to withstand limitation in life supporting oxygen or nutrient supply. Many of the genetic defects found in tumors reside in signaling cascades or proteins, supporting the key role of aberrant signaling in this. However, protective mechanisms also can be triggered in a more rapid and transient fashion. During transplantation organs are temporarily deprived of oxygen, nutrients and growth factors (ischemia). A decrease in myocardial oxygen delivery initially results in decreased contractile activity and oxygen consumption, a phenomenon termed *hibernating myocardium *[1]. Prolonged hypoxia may also reduce ATP demand by down-regulating protein synthesis [2]. Restoration of blood flow (reperfusion) corrects these deficits but at the same time initiates a detrimental cascade, which in the end can lead to irreversible damage of the affected organ. During reperfusion organs also respond with the activation of intracellular signaling pathways. Some of these signaling activities are clearly linked to the cell death induction, while others may be involved in the elicitation of a protective response. Through repeated short ischemic intervals which do not cause irreversible damage ("pre-conditioning"), cells may actually learn to cope with conditions, which are normally life-threatening. Also this defense mechanism is operated by intracellular signaling pathways [3]. Therefore, understanding the role of signaling in ischemia-reperfusion induced injury (IRI) holds the potential for the development of novel therapeutic strategies for its prevention, perhaps even at a time point before other events like the production of reactive oxygen species (ROS), the perturbation of Ca^2+ ^homeostasis or inflammatory responses occur, which will greatly amplify the damage. Mitochondria feature prominently in these processes as they are critical for the provision of energy and the assurance of cell survival. A coordinated cellular response will require crosstalk between mitochondria and their cellular environment. Cytoplasmic signaling pathways are increasingly recognized for their central role in relaying information to the mitochondria and in possibly controlling their function [4]. In this review we will present a current view of mitochondrial form and function, before we discuss the dealings of mitochondria with the cellular environment. Finally we will use the example of ischemia/reperfusion-induced tissue damage to illustrate the cooperation of these players and to show up possible therapeutic implications.

## From form to function: Mitochondria shaping up for performance

The classical view of mitochondria as bean-shaped organelles has changed dramatically over the last years. Earlier studies shed light on the complexity of their internal organization [5], while most recently another feature of these organelles has attracted considerable interest, their highly dynamic behavior [6]. Through fission and fusion mitochondrial morphology can change from small spheres or short rods to long tubules forming large network-like structures within the same cell. Another level of complexity is added through their high motility. While the highest velocity was found in neurons, mitochondrial movement could be shown in various other cell systems such as HL-1 cells, cultured fibroblasts, budding yeast, etc. [7-11]. Mitochondria can be actively transported in cells and may have defined tissue-specific subcellular distributions. In neurons, for example, they are translocated to regions with high energy and Ca^2+ ^buffering demands, such as active growth cones, pre- and post-synaptic sites. In addition, they often pause at sites where no mitochondria are present, resulting in an uniform axonal mitochondrial distribution. Mitochondria with high membrane potential preferentially migrate in the anterograde direction, whereas mitochondria with low membrane potential move in the retrograde direction. Therefore active mitochondria appear at distal regions with high energy demands, while impaired mitochondria are returned to the cell soma, possibly for repair or mitophagy. In addition, signaling molecules such as nerve growth factor (NGF) influence mitochondrial recruitment and retention [12].

Several studies have suggested that controlling mitochondrial shape through fusion and fission is also crucial for maintaining the functional properties of mitochondria [6]. The precise balance between these two opposing processes therefore might play a key role in mitochondrial and cellular function. More than ten years ago, the first molecular mediator of mitochondrial fusion was discovered in *Drosophila melanogaster*: Fusion factor *fuzzy onions *(Fzo), a mitochondrial outer membrane GTPase that is required for the fusion of mitochondria during spermatogenesis [13]. The two human Fzo homologues Mitofusin (Mfn) 1 and 2 also control mitochondrial morphology [14,15]. As a subset of mitochondria in Mfn1-deficient cells was shown to lose their membrane potential, mitochondrial fusion seems to allow cooperation between mitochondria, thereby protecting mitochondria from respiratory dysfunction [15]. Also Mfn2 ablation experiments have documented a reduced mitochondrial oxidation and membrane potential as well as a repression of nuclear-encoded subunits of OXPHOS complexes [16]. In mammals, mitochondrial fission requires the recruitment of cytosolic dynamin-related protein (DRP1) [17]. Post-translational modifications regulate its function in mitochondrial fission. The ubiquitin ligase of the mitochondrial outer membrane, termed membrane-associated RING-CH (MARCH)-V, a member of the transmembrane RING-finger protein family, has the ability to bind to Mfn2 and DRP1, to ubiquitinate DRP1 and to modify mitochondrial morphology. By regulating Mfn2 and Drp1 activities, MARCH-V controls mitochondrial morphology [18].

In addition, cellular stress influences mitochondrial fusion and fission: Thus DRP1 was also shown to be involved in apoptosis-associated mitochondrial fission. While under normal conditions DRP1 recycles between the cytoplasm and mitochondria, it shows a stable membrane association following the recruitment of BAX to the mitochondrial membrane but before the loss of mitochondrial membrane potential. During apoptosis, the biochemical properties of DRP1 are regulated via a BAX/BAK-dependent stimulation of small ubiquitin-like modifier-1 conjugation to DRP1 [19]. Recent findings have demonstrated a key role of the proper regulation of mitochondrial dynamics for various cellular pathways, demonstrating also that defective mitochondrial behavior may affect human health. For example, several neurological diseases (e.g. Charcot-Marie-Tooth, CMT; autosomal dominant optic atrophy, DOA) are associated with mutations in proteins that control mitochondrial dynamics and morphology like mitofusin-2 and OPA1 [20,21].

## Breathing oxygen-producing ROS

The change in earth's atmosphere from anoxic to our current conditions of approx. 21% oxygen had on the one hand catastrophic effects on unicellular anaerobic life forms but on the other hand paved the way for new forms of life which managed to tame this highly reactive and due to its oxidizing properties potentially life-threatening molecule and made a renegade of it changing fronts from life threatening to life supporting [22]. The supply of ATP through oxidative phosphorylation, which couples the oxidation of metabolic substrates to the synthesis of ATP from ADP and inorganic phosphate takes place in the mitochondria, putative descendents of an ancient endosymbiotic event between an alpha-proteobacterium and an archean host [23,24]. Utilizing oxygen as the final electron acceptor in the aerobic metabolism of glucose as the primary source of energy increased the efficiency of ATP generation.

The mitochondrial respiratory chain transfers electrons to molecular oxygen, permanently producing ROS as a by-product of oxidative phosphorylation. Depending on the conditions, a few percents of the oxygen consumed by mitochondria are reduced by a single free electron with formation of superoxide radical, which then can be converted to hydrogen peroxide (further mono-electron reduction of oxygen) by mitochondrial superoxide dismutase (SOD), which in turn can be scavenged by catalase reaction or converted to the very reactive hydroxyl radical in the presence of transition metals (e.g. Fe^2+^). Several reports have demonstrated that mitochondrial superoxide production is mostly a result of incomplete reduction of oxygen at sites of respiratory complexes I (NADH:ubiquinone oxidoreductase) and III (CoQ:cytochrome *c *oxidoreductase) which therefore can be considered as main sources for mitochondrial ROS [25-29].

## Sensing cellular energy status

Mitochondria are able to monitor their surrounding environment, including intracellular energy (ATP) levels, as well as oxygen, ROS, Ca^2+ ^and the presence or absence of growth factors [4]. Moreover, mitochondria are well tailored to meet both the signaling and metabolic needs of the cell. It has been suggested that mitochondrial biogenesis (mitochondrial proliferation) and dynamics are strongly linked to the ability of mitochondria to sense energy status [7,30-33]. In muscles confronted with increased work load (training, endurance exercise) or pathological changes (mitochondrial diseases, genetic defects in respiratory complexes), the proliferation of mitochondria serves as an adaptational response to decreased energy levels [33]. In other cells like neurons, energy sensing mechanisms may also serve for the directorial transport of mitochondria to the cellular regions of higher energy demands [7]. One of the key enzymes for low-energy sensing is AMP activated protein kinase (AMPK) [34]. AMPK is an evolutionarily conserved enzyme which is allosterically activated by AMP (marker of low energy status). In addition, AMPK is strongly regulated by changes in phosphorylation state by upstream kinases and phosphatases [34-36]. This enzyme therefore is sensitive to increases in the cellular AMP/ATP ratio and can be activated by various metabolic stresses, such as ischemia, hypoxia, starvation (e.g. glucose deprivation), by metabolic inhibition (e.g. using analogs of energy substrates or simulated ischemia) or in response to increased exercise in muscles [31,37-40]. Stimuli for AMPK involve either processes that inhibit ATP production or accelerate ATP consumption. Active AMPK upregulates catabolic and suppresses anabolic pathways [38,41]. For example, AMPK may phosphorylate and thus inhibit enzymes of ATP consuming pathways like the formation of fatty acids, cholesterol and glycogen (e.g. acetyl-CoA-carboxylase, glycogen synthase, etc.), and also highly ATP consuming protein synthesis by eEF2K phosphorylation [42]. Moreover, active AMPK blocks cell growth and proliferation by suppression of the target of rapamycin (mTOR) pathway [43,44] via direct phosphorylation of an upstream regulator of mTOR, tuberous sclerosis complex-2 (TSC2 or tuberin) [45]. This type of downregulation of mTOR signaling seems to be dominant over the positive effects of growth factors or amino acids. More recent data suggest two inhibitory effects of AMPK on mTOR. Activated AMPK may phosphorylate TSC2 at a site different from AKT, promoting its Rheb-GAP activity, and additionally it may phosphorylate raptor (regulatory associated protein of mTOR), also resulting in mTOR inhibition [46], leading to a suppression of protein synthesis and overall cellular ATP consumption. At the same time, AMPK activation in muscles could markedly stimulate glucose uptake by increased translocation of the glucose transporter GLUT4 to the plasma membrane [47] via phosphorylation of a downstream target of AMPK – AS160, a Rab GTPase-activating protein [48]. Besides, acute or chronic chemical activation of AMPK in muscles results also in elevated GLUT4 expression (with remarkable increase in GLUT4 mRNA levels) [49,50]. Therefore, through activation of both GLUT4 translocation and GLUT4 expression, activated AMPK stimulates glycolysis in muscles. In addition, activation of 6-phosphofructo-2 kinase (PFK) by AMPK also supports glycolytic ATP production. Furthermore, phosphorylation of acetyl-CoA carboxylase (ACC) by AMPK decreases malonyl-CoA levels [51], reducing inhibition of carnitine palmitoyl-CoA acyltransferase-1 (the enzyme responsible for transport of fatty acids into mitochondria), stimulating utilization of fatty acids and helping thus better mitochondrial ATP production. AMPK activation therefore serves to defend against energy deficiency via activation of glucose transport and oxidation of fatty acids [34,52-54]. Growing evidence [30,31] demonstrates that AMPK is also a critical regulator involved in initiating mitochondrial biogenesis through activation of the peroxisome proliferator activated receptor γ coactivator 1α (PGC-1α) which is an important regulator of transcription of many genes involved in mitochondrial energy metabolism, mitochondrial physiology and oxidation of glucose and fatty acids [55]. Notably, AMPK also interferes with mitochondrially produced ROS and reactive nitrogen species (RNS), as well as with their scavengers: like vitamin E, N-acetylcysteine, the SOD-mimetic MnTBAP, or α-Lipoic acid (powerful antioxidant and an essential cofactor for several mitochondrial enzymes) [40,56,57].

Metabolic dysregulation is commonly observed under conditions of metabolic stress, e.g. cancer, ischemia/reperfusion. In tumors it may be an important contributor to the transformation process. Thus the frequently described switch to glycolysis which also persists under aerobic conditions ("Warburg effect") may in part be caused by direct effects of cancer protein signaling on the expression/activity of glycolytic enzymes [58-60]. The discovery of mutations in succinate dehydrogenase and fumarate hydratase, components of the tricarboxylic acid (TCA) cycle (also known as Krebs cycle), which connects cytosolic glucose metabolism to mitochondrial oxidative phosphorylation (OXPHOS), led to the demonstration of a tumor suppressor function for these proteins [61]. When these genes are mutated, succinate or fumarate, respectively, accumulate in mitochondria and pass to the cytosol resulting in the inhibition of prolyl hydroxylases (PHDs) and consecutive stabilization of transcription factor hypoxia-inducible factor 1α (HIF1α) under normoxic conditions [62] with important consequences for the expression of target genes required for tumor growth and metastasis. Finally, also mitochondrial DNA (mtDNA) may carry mutations which through impairment of OXPHOS, increased ROS production and increased proliferation contribute to tumor progression [63].

## Controlling mitochondrial function by modulating intracellular signaling

Ischemia and reperfusion cannot be avoided during organ transplantation and initiate a cascade of events, which results in tissue damage. While advances in immunosuppressive therapy, amelioration of surgical techniques and organ preservation have significantly improved success rates of solid organ transplantation, IRI remains a major problem requiring substantial follow up treatment [64-66].

Massive mitochondrial ROS production during reperfusion paralleled by the depletion of scavengers like superoxide dismutase (SOD), vitamins C and E etc. results in the deterioration of organ function or even organ loss [67-69]. There is also evidence that crucial events leading to ROS production already occur during ischemia [70]. Nevertheless, the major hit to the oxygen-deprived cell happens, paradoxically, during reperfusion. The reperfused cells experience an "oxidative burst" with mitochondria-derived superoxide radicals [71,72]. Mitochondria are especially sensitive to ROS induced damage and as a consequence disruption of oxidative phosphorylation can be observed culminating in significant reduction of ATP levels, excessive entry of Ca^2+ ^into mitochondria and loss of mitochondrial membrane potential [73,74], resulting in cytosolic release of apoptosis inducing factors, such as apoptosis inducing factor (AIF), cytochrome *c *and Smac/DIABLO [75,76].

**Figure 1:**
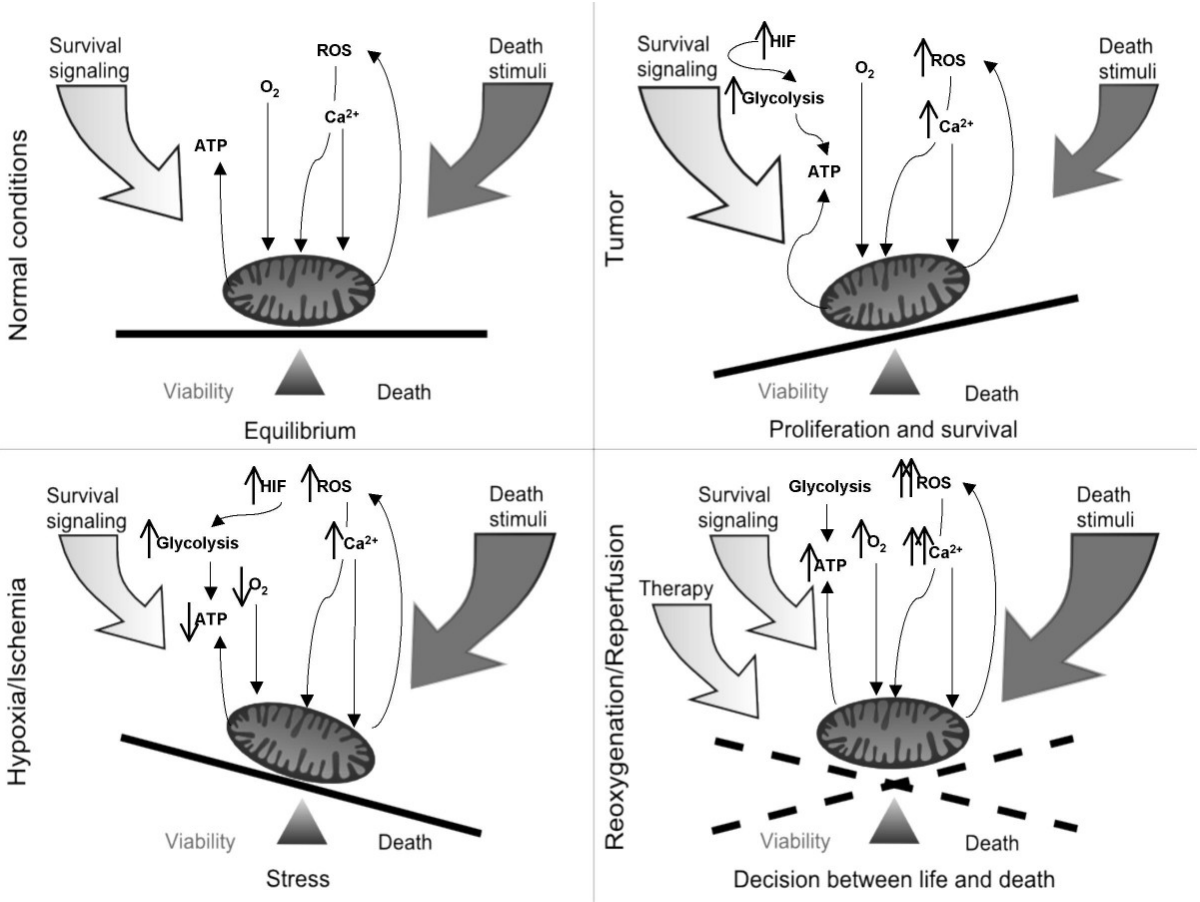
**Mitochondrial rheostat of cell survival control**. Key players in the regulation of mitochondrial function are depicted under four different states. Intracellular signaling is represented by bold arrows, filled light grey for survival or dark grey for pro-apoptotic signaling. Under normal conditions cells tolerate a certain level of stress before strong pro-apoptotic stimuli eventually perturb cellular homeostasis. A tumor cell no longer is in a state of equilibrium as ROS levels usually are high, survival signals are enhanced and extra-mitochondrial energy production may become predominant. During ischemia/hypoxia oxidative phosphorylation is disturbed leading to a decrease in ATP. Additionally, cells suffer from elevated ROS and Ca^2+ ^levels. During reperfusion/reoxygenation mitochondrial ROS and Ca^2+ ^levels peak and cell death may follow. Various intracellular signaling pathways are activated during this time and may positively or negatively control mitochondrial function. Eventually, a therapy that abolishes pro-apoptotic signals or elicits survival signaling may help to prevent or limit damage occurring under these conditions.

Attempts to limit ischemia/reperfusion-associated cellular damage have to take into account the important role of mitochondria in this process. Current strategies try to limit the extent of ROS damage by applying anti-oxidants. Much more desirable would be an approach, which avoids oxidative damage by preventing ROS production or scavenging oxygen radicals at the site of their production. First evidence for the control of mitochondrial events by cellular signaling pathways was provided by demonstrating their effect on the expression and function of anti-apoptotic proteins of the Bcl-2 or IAP family [77]. Additional support came from the suggested localization of many diverse signaling molecules (kinases, transcription factors, etc.) to various sites in the mitochondria [4,78]. More difficult was the search for targets regulated by them due to experimental difficulties. Candidate processes controlled through signaling include protein and Ca^2+ ^trafficking, oxidative phosphorylation and production of reactive oxygen species. A critical event in cell death initiation is the translocation of the pro-apoptotic Bcl-2 protein BAX to the mitochondria [79,80]. Cessation of survival signals, which is a common stimulus for cell death induction, will result in the shut-down of signaling cascades, and in particular the kinases, which compose them. Since phosphorylation may both positively and negatively regulate protein function, signal interruption also will cause the generation of new signals. One of them originates from GSK-3, which upon activation will cause the phosphorylation-dependent ubiquitination and subsequent degradation of the survival protein Mcl-1, which normally negatively controls BAX, followed by its mitochondrial translocation [81]. In our work we recently demonstrated that expression of oncogenic or wild type RAF prevented mitochondrial ROS production, Ca^2+ ^overload and apoptosis [82]. Protein kinase A (PKA) has been implicated in the activation of the NADH-ubiquinone oxidoreductase activity of complex I resulting in reduced ROS production [83,84]. Decreased mitochondrial ROS levels were also observed in the heart of transgenic mice expressing the p38 MAPK activator MAPK kinase 6 (MKK6) [85]. Also the tumor suppressor p53 can control ROS levels through its transcriptional target TIGAR [86], leading to an increase in the levels of glutathione (GSH), which scavenges ROS. In contrast, increased mitochondrial ROS production has been described for SHC [87,88]. A fraction of p66 (SHC) exists within mitochondria, where it oxidizes cytochrome *c *to form hydrogen peroxide, which in turn induces mitochondrial permeability and apoptosis. Taken together these examples demonstrate that key mitochondrial processes can be subject to the regulation by signaling pathways, which normally respond to extrinsic stimuli (Figure 1). Many of the signaling molecules have attracted considerable interest in the past because of their role in diverse pathological settings including autoimmune diseases, inflammation or cancer. Various approaches have been developed to target them for therapeutic purposes including the development of small molecular weight inhibitors. This raises the possibility of planned pre-emptive intervention to also limit the extent of IRI.

Production of mitochondrial ROS is not only restricted to IR but indeed may be an important intermediate in intrinsic and extrinsic (cell death receptor-dependent) pathways of cell death induction. Thus growth factor abrogation [82,83], death induction through activation of the TNF-α receptor [89,90] or genotoxic stress [91,92] all are linked to the induction of massive ROS production, which is essential for cell death.

## Conclusion

Mitochondria long have been recognized for their role as powerhouse of the cell. Interest in mitochondria was greatly rekindled upon recognition of their central role in regulation of cell death. Many death stimuli converge on these organelles to cause release of apoptogenic factors. This mitochondrial response is also coupled to the interruption of energy production and a collapse of mitochondria ROS and Ca^2+ ^homeostasis. However, the latter processes may also be directly targeted by pro- and antiapoptotic signaling pathways (Figure 1) opening up possible novel options for therapeutic interference at an early stage, before through the release of second messenger like ROS the damage to cells and organs is amplified.

## Competing interests

The authors declare that they have no competing interests.

## Authors' contributions

JT planned the review outline, drafted the final version. All the other authors equally contributed to the writing of the review.
